# Chronic treatment with acetaminophen protects against liver aging by targeting inflammation and oxidative stress

**DOI:** 10.18632/aging.202884

**Published:** 2021-03-29

**Authors:** Rocío Brea, Pilar Valdecantos, Patricia Rada, Rosa Alen, Carmelo García-Monzón, Lisardo Boscá, Marina Fuertes-Agudo, Marta Casado, Paloma Martín-Sanz, Ángela M. Valverde

**Affiliations:** 1 Instituto de Investigaciones Biomédicas “Alberto Sols”, (CSIC-UAM), Department of Metabolism and Cellular Signaling, Madrid 28029, Spain; 2 Centro de Investigación Biomédica en Red de Diabetes y Enfermedades Metabólicas Asociadas (CIBERdem) ISCIII, Madrid 28029, Spain; 3 Liver Research Unit, Hospital Universitario Santa Cristina, Instituto de Investigación Sanitaria Princesa, Madrid 28009, Spain; 4 Centro de Investigación Biomédica en Red de Enfermedades Cardiovasculares (CIBERcv) ISCIII, Madrid 28029, Spain; 5 Instituto de Biomedicina de Valencia (IBV-CSIC), Valencia 46010, Spain; 6 Centro de Investigación Biomédica en Red de Enfermedades Hepáticas y Digestivas (CIBERehd) ISCIII, Madrid 28029, Spain

**Keywords:** APAP, aging, inflammation, liver, oxidative stress

## Abstract

The liver exhibits a variety of functions that are well-preserved during aging. However, the cellular hallmarks of aging increase the risk of hepatic alterations and development of chronic liver diseases. Acetaminophen (APAP) is a first choice for relieving mild-to-moderate pain. Most of the knowledge about APAP-mediated hepatotoxicity arises from acute overdose studies due to massive oxidative stress and inflammation, but little is known about its effect in age-related liver inflammation after chronic exposure. Our results show that chronic treatment of wild-type mice on the B6D2JRcc/Hsd genetic background with APAP at an infratherapeutic dose reduces liver alterations during aging without affecting body weight. This intervention attenuates age-induced mild oxidative stress by increasing HO-1, MnSOD and NQO1 protein levels and reducing ERK1/2 and p38 MAPK phosphorylation. More importantly, APAP treatment counteracts the increase in Cd8^+^ and the reduction in Cd4^+^ T lymphocytes observed in the liver with age. This response was also found in peripheral blood mononuclear cells. In conclusion, chronic infratherapeutic APAP treatment protects mice from age-related liver alterations by attenuating oxidative stress and inflammation.

## INTRODUCTION

The liver is a pivotal organ with an extensive variety of functions, including drug metabolism and detoxification, protein synthesis, and regulation of energy metabolism, among others [1]. Most liver functions appear to be well preserved with age, but some changes in liver morphology and physiology, such as enhanced hepatocyte size, increased number of binucleated cells, and reduction in mitochondrial number [2], have been associated with increased risk of hepatic injury and development of many chronic liver diseases [3]. Inflammation is of particular interest, since aging is characterized by increased levels of a number of pro-inflammatory molecules including acute phase proteins (CRP and serum amyloid A), cytokines (TNF-α, IL-6, and IL-8), and adhesion molecules (sICAM-1 and sVCAM-1) in circulation, a phenomenon that has been termed “inflammaging” [4–7]. It is also hypothesized that failure of anti-inflammatory and inflammation resolving mechanisms contributes to chronic inflammation in the elderly. In this context, aging is associated with substantial changes in both innate and adaptive immune systems in order to counteract functional defects of Cd8^+^ T lymphocytes [8, 9] and the decrease in the function of Cd4^+^ T helper cells [10]. Moreover, recent advances point to natural killer (NK) and natural killer T (NKT) cells as a bridge between the innate and adaptive immune systems [11]. Particularly, the role of the liver as a reservoir of pluripotent stem cells, which give rise to multiple cell lineages, including NKT [12, 13] and Cd8^+^ T cells [14], and the adaptive immune response during chronic non-alcoholic fatty liver disease (NAFLD) has been the focus of recent research as reviewed [15]. Thus, slow or reverse the process of inflammaging is highly relevant to achieve a healthy aging and improve well-being.

According to many international guideline recommendations, acetaminophen (N-acetyl-p-aminophenol, paracetamol, APAP) is a first choice drug for relieving mild-to-moderate pain based on its analgesic and antipyretic effects [16]. At therapeutic doses (up to 4 g per day in adult humans), APAP represents a valuable agent in the pharmacological management of pain, especially for patients who cannot tolerate non-steroid anti-inflammatory drugs such as aspirin, ibuprofen or salicylates, which can cause detrimental effects on the gastrointestinal tract and kidney [17]. At non-toxic doses, APAP is rapidly metabolized in the liver through glucuronidation and sulfation. However, a small part is also oxidized by cytochrome P450 isoforms (mainly CYP2E1, CYP3A4, and CYP1A2) generating the toxic metabolite N-acetyl-p-amino benzoquinone imine (NAPQI). This intermediate is highly reactive and cytotoxic. At therapeutic doses, NAPQI is detoxified by glutathione (GSH) and eliminated in urine or bile as APAP-cysteine, APAP-N-acetyl cysteine, and APAP-GSH. On the contrary, after an overdose glucuronidation and sulfation pathways become saturated triggering a rapid depletion of the hepatic GSH pool. As a result, massive amounts of NAPQI bind to the cysteine groups of proteins and generate APAP-protein adducts leading to oxidative stress and inflammation [17].

Hitherto, most of the knowledge about APAP-mediated hepatotoxicity arises from studies describing its toxic effect upon APAP overdose in experimental mouse models or humans [18–20]. Our recent data revealed that sirtuin 1 (SIRT1) overexpression confers protection against acute APAP-mediated hepatotoxicity by targeting IL1β/NF-κB-mediated inflammation [21]. However, little is known about the effect of APAP after a chronic or infratherapeutic exposure in age-related liver inflammation.

Taking these into account, the present study aims to investigate in detail the effects of chronic treatment of mice with an infratherapeutic dose of APAP and its relationship with age-related oxidative stress and inflammation in the liver.

## RESULTS

### An infratherapeutic dose of APAP has a mild beneficial effect in hepatic parameters altered during aging without affecting body weight

**Figure 1:**
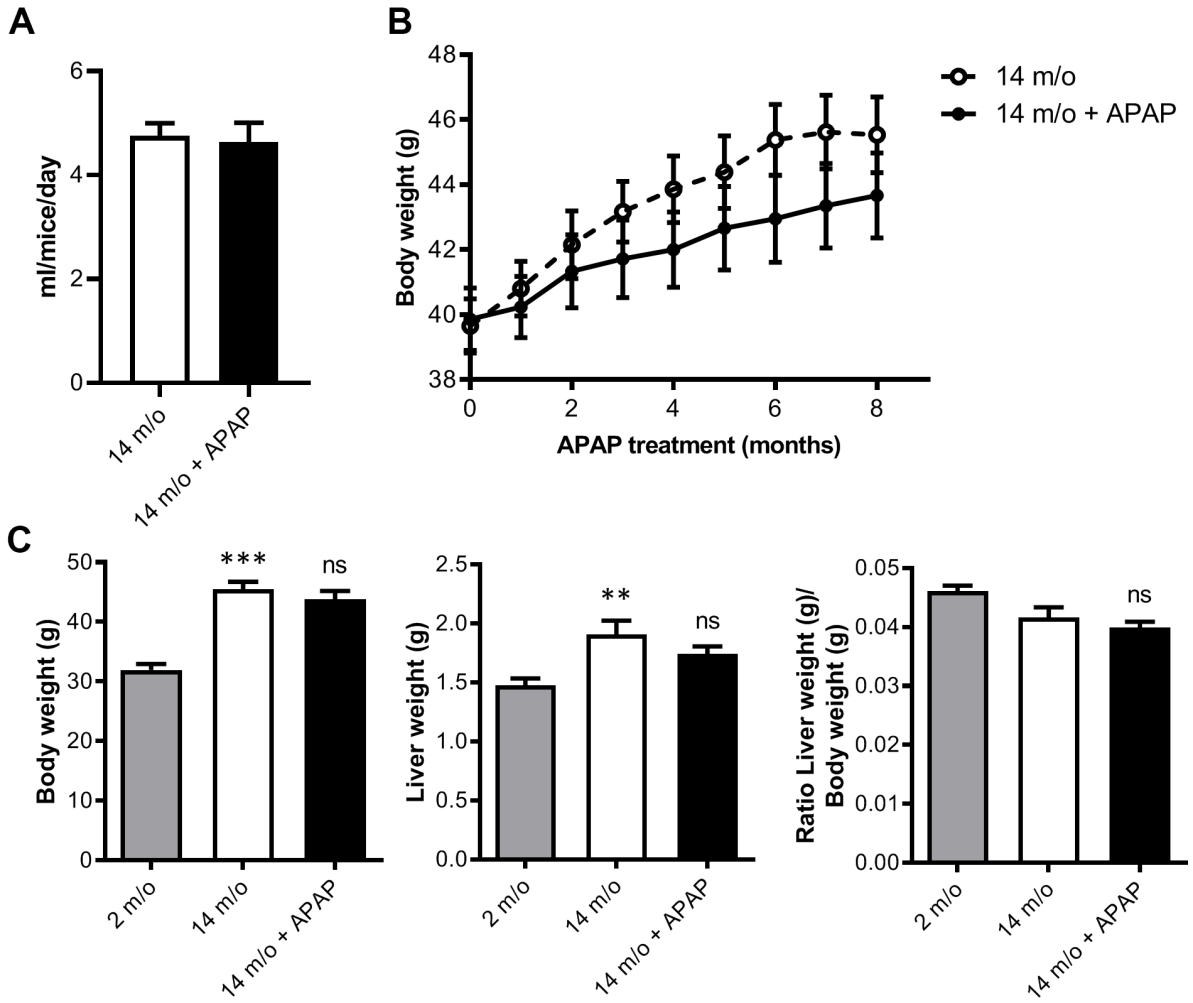
**Chronic APAP treatment at an infratherapeutic dose does not change body and liver weight.** (**A**) Mean daily water consumption. (**B**) Body weight of mice throughout 8 months of APAP treatment. (**C**) Comparison of the body weight, liver weight and liver-to-body weight ratio of 2 m/o, 14 m/o and 14 m/o + APAP mice. Data are represented as the mean ± S.E.M. (n = 17-21 mice per group). Statistical analysis was performed by Mann-Whitney test (**A**), ANOVA test for repeated-measures (**B**) and Kruskal-Wallis or Brown-Forsythe and Welch ANOVA test followed by their respective post-hoc test (**C**). ** *P* < 0.01 and *** *P* < 0.001 *vs*. 2 m/o; ns *vs*. 14 m/o.

To investigate the effects of chronic APAP treatment and its relationship with aging-mediated deleterious effects in the liver, mice were treated with an infratherapeutic dose of APAP dissolved in the drinking water for 8 months as detailed in “Methods” section. We used wild-type mice on the B6D2JRcc/Hsd genetic background as we previously demonstrated that they are susceptible to diet-induced metabolic and liver damage [22, 23]. APAP treatment did not induce any abnormal clinical signs during the study. As shown in Figure 1A, the mean daily water consumption did not significantly differ between groups regardless of APAP supplementation. Furthermore, APAP treatment did not result in any significant change in the group mean body weight and mean cumulative net body weight gain when compared to the untreated control group (Figure 1B). In order to not only analyze the impact of APAP, but also the effect of age, a cohort of two-month-old mice (2 m/o) was included. As expected, total body and liver weight observed in 14 m/o mice was significantly higher than that of the 2 m/o group and also liver-to-body weight ratio was reduced (Figure 1C). However, APAP treatment did not alter the changes associated with age.

To assess the impact of *in vivo* chronic APAP exposure on glucose homeostasis, GTT was performed in all groups at the end of the treatment. As shown in Supplementary Figure 1A, 1B, no significant differences were found in glucose tolerance regardless of aging or APAP treatment. To study the specific effect of APAP treatment in the liver, PTT was conducted (Supplementary Figure 1C, 1D) and, in the same line, we did not find differences among experimental groups although a non-significant delayed response was observed in mice receiving APAP. Thus, our results demonstrated that in these cohorts of mice neither age nor APAP treatment impaired whole-body glucose homeostasis.

**Figure 2:**
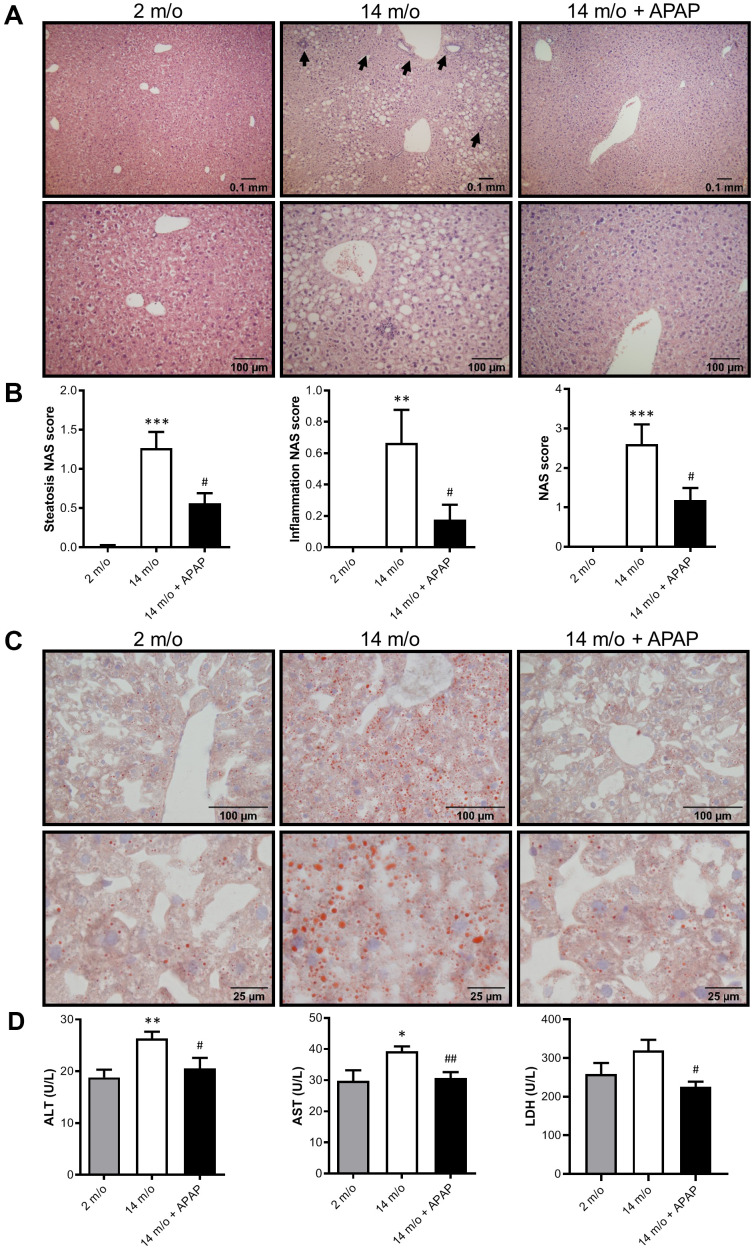
**Chronic APAP exposure ameliorates the effect of age in liver alterations.** (**A**, **C**) Representative images (10X and 20X) of H&E (**A**) and Oil Red O (**C**) staining performed on various sections of livers from 2 m/o, 14 m/o and 14 m/o + APAP mice. (**B**) Steatosis NAS score, inflammation NAS score, and total NAS score. (**D**) Plasma ALT, AST and LDH levels from 2 m/o, 14 m/o and 14 m/o + APAP mice. Values are expressed in international units per liter (U/L). Data are represented as the mean ± S.E.M. (n = 6-18 mice per group). Statistical analysis was performed by one-way ANOVA or Brown-Forsythe and Welch ANOVA test followed by their respective post-hoc test. * *P* < 0.05, ** *P* < 0.01 and *** *P* < 0.001 *vs*. 2 m/o; ^#^ *P* < 0.05 and ^##^ *P* < 0.01 *vs*. 14 m/o.

Next we addressed whether chronic treatment with an infratherapeutic dose of APAP induces liver alterations. To analyze histological damage, liver sections were stained with H&E and evaluated by a hepatopathologist. As shown in Figure 2A, 2B, both steatosis and inflammation induced by aging were significantly attenuated by the treatment with APAP. Consequently, a reduced NAS score was observed in 14 m/o + APAP group. The results obtained after staining liver sections with Oil Red O (Figure 2C) agree with these data, showing less lipid accumulation in APAP-treated 14 m/o mice when compared to the 14 m/o group. In addition, evidence of aging-associated liver changes was detected in 14 m/o mice as reflected by the analysis of plasma transaminases (ALT, AST) (Figure 2D) that showed a moderate increase in agreement with other studies [24, 25]. More importantly, in APAP-treated mice, plasma ALT, AST and lactate dehydrogenase (LDH) levels were similar to those of 2 m/o (Figure 2D) indicating that chronic APAP exposure ameliorates age-related liver dysfunction.

### Chronic APAP administration attenuates age-induced mild oxidative stress in mice

**Figure 3:**
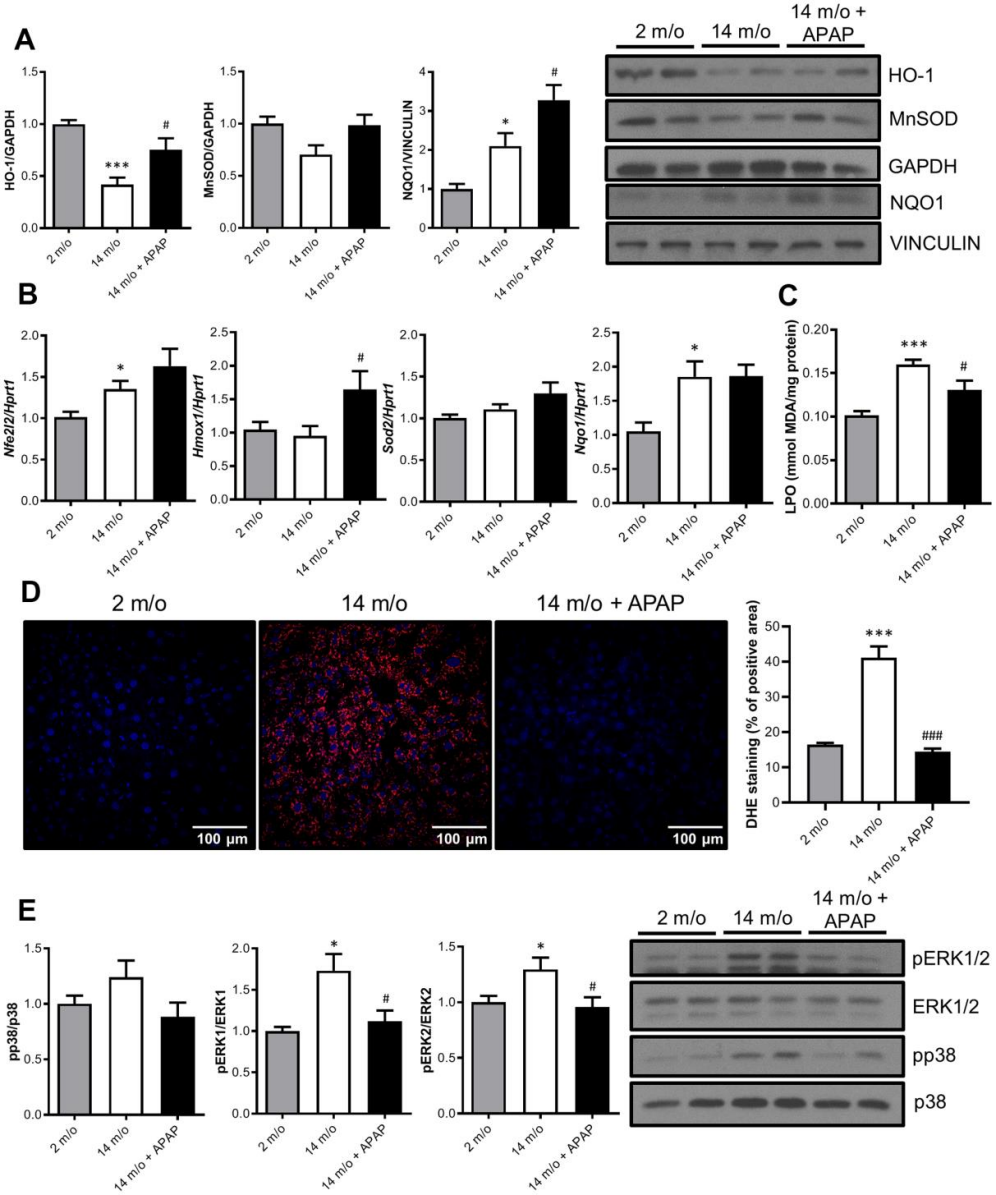
**Chronic APAP administration at an infratherapeutic dose increases Nrf2-targets HO-1, NQO1 and MnSOD and enhanced antioxidant capacity.** (**A**) Representative Western blots showing HO-1, MnSOD and NQO1 protein levels in liver extracts from 2 m/o, 14 m/o and 14 m/o + APAP mice. GAPDH and VINCULIN levels were used as loading controls (n = 6-14 mice per group) (see original western blot in Supplementary Figure 6). (**B**) Liver mRNA expression of *Nfe2l2, Hmox1, Sod2* and *Nqo1* from 2 m/o, 14 m/o and 14 m/o + APAP mice, analyzed by qRT-PCR. Values have been normalized against *Hprt1* mRNA, and expressed as fold of change *vs*. 2 m/o (n = 6-8 mice per group). (**C**) Hepatic LPO levels of 2 m/o, 14 m/o and 14 m/o + APAP mice (n = 6-8 mice per group). (**D**) Detection of O_2_^•−^ in frozen liver sections from 2 m/o, 14 m/o and 14 m/o + APAP group mice using DHE (n = 5 mice per group). (**E**) Representative Western blots showing phospho-ERK1/2 and phospho-p38 MAPK protein levels. Total ERK1/2 and p38 MAPK levels were used as loading controls, respectively (n = 9-14 mice per group) (see original western blot in Supplementary Figure 7). For (**A**, **E**), graphs depict densitometric quantifications of the indicated protein levels. Data are represented as the mean ± S.E.M. Statistical analysis was performed by Kruskal-Wallis, one-way ANOVA or Brown-Forsythe and Welch ANOVA test followed by their respective post-hoc test. * *P* < 0.05 and *** *P* < 0.001 *vs*. 2 m/o; ^#^ *P* < 0.05 and ^###^ *P* < 0.001 *vs*. 14 m/o.

It is classically argued that oxidative stress may be one of the causal factors underlying the aging process due to a reduction of the antioxidant defenses [26]. Since long-term APAP administration ameliorates age-associated liver changes, we next explored the antioxidant signature in the liver. Results depicted in Figure 3A revealed that protein levels of two-well known antioxidant enzymes, HO-1 and MnSOD, were reduced with age whereas an unexpected increase in NQO1 was found. Importantly, APAP treatment reinforced the antioxidant response of the liver by increasing HO-1, MnSOD and NQO1 protein levels. In agreement with these data, APAP also promoted a slight increase in mRNA levels of *Nfe2l2* (encoding NRF2) and its target genes *Hmox1* and *Sod2* (Figure 3B). In contrast to the protein levels, *Nqo1* mRNA levels did not show differences after 8-months APAP treatment. Also, no differences were found in nuclear NRF2 protein levels (Supplementary Figure 2). Furthermore, the antioxidant status was evaluated by measuring lipid peroxidation (LPO) and cellular O_2_^•−^ content in the liver. As shown in Figure 3C, APAP treatment attenuated age-induced LPO levels. Also, DHE-derived fluorescence analyzed in liver sections revealed that whereas aging increased O_2_^•−^ production, treatment with an infratherapeutic dose of APAP significantly reduced O_2_^•−^ content (Figure 3D). Altogether, our results strongly suggest that chronic APAP administration at an infratherapeutic dose in mice favored the preservation of an appropriate antioxidant/oxidant balance in the liver, thereby maintaining healthy hepatic aging.

To date, it is well-recognized that MAPKs are activated in response to cell stressors such as oxidative and inflammatory triggers and have been implicated in different modes of hepatocyte death [27]. Indeed, numerous studies have proven the contribution of JNK, p38 MAPK, and ERK1/2 during acute APAP-induced liver toxicity [27–29]. However, little is known about their contribution after chronic exposure to infratherapeutic doses of this drug. As shown in Figure 3E, an increase in p38 MAPK and ERK1/2 phosphorylation levels was observed with age. Moreover, in mice receiving chronic APAP administration, a decrease in the phosphorylation of both kinases was found; being differences in pERK1/2 levels statistically significant, and suggesting a protective anti-aging effect of this drug. Likewise, under these conditions we were unable to detect changes in the phosphorylation of JNK1/2 in livers from 2 m/o or 14 m/o mice regardless of the APAP treatment (data not shown).

Previous results showed that after an APAP overdose activation of NF-κB-mediated signaling triggers oxidative stress and a pro-inflammatory response, ultimately causing liver damage [21, 30, 31]. To determine whether NF-κB signaling is modulated in the liver by chronic and infratherapeutic APAP administration, nuclear p65-NF-κB and cytosolic IκBα protein levels were analyzed. As shown in Supplementary Figure 2, no significant changes were found regardless of aging or treatment, ruling out the involvement of the NF-κB pathway in the liver neither in aging nor in the protective effect mediated by chronic APAP administration.

### Impact of aging and chronic treatment with APAP in the immune signature of the liver

**Figure 4:**
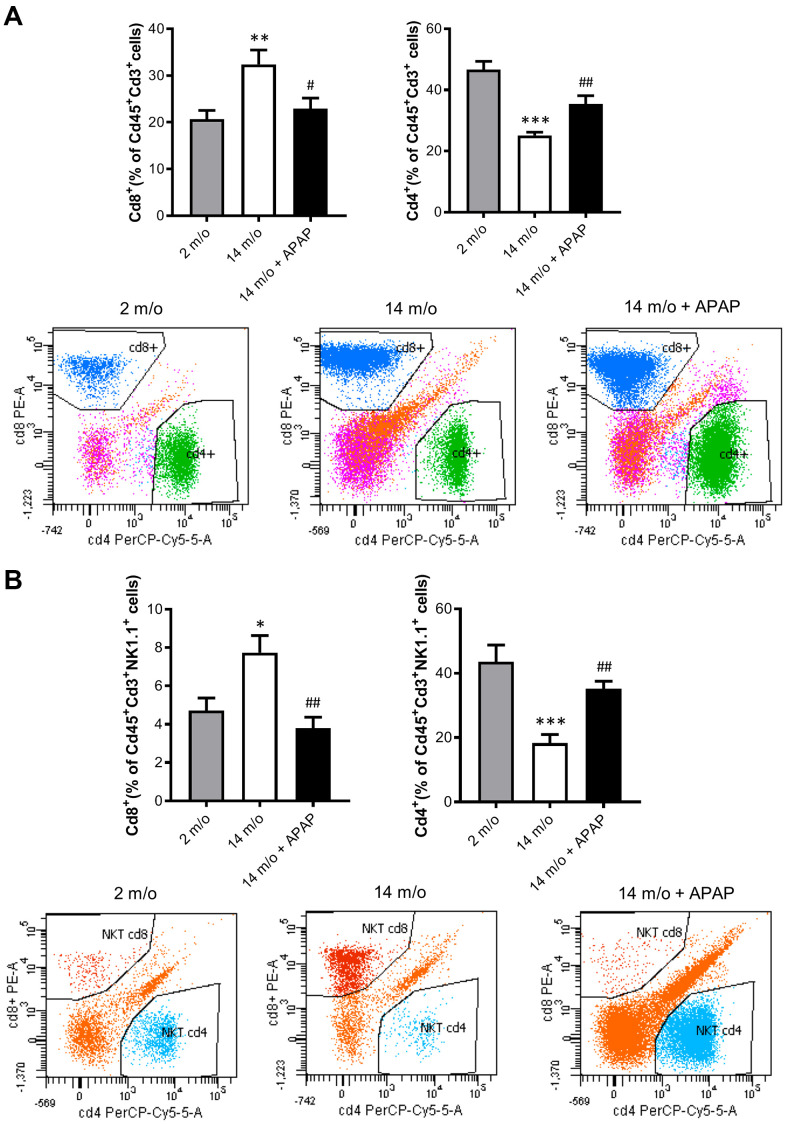
**Analysis of lymphoid populations in liver NPCs from 2 m/o, 14 m/o and 14 m/o + APAP mice by flow cytometry.** (**A**) Percentage of Cd8^+^ (left) and Cd4^+^ (right) from Cd45^+^Cd3^+^ cells and representative images of flow cytometry analysis. (**B**) Percentage of Cd8^+^ (left) and Cd4^+^ (right) from Cd45^+^Cd3^+^NK1.1^+^ cells and representative images of flow cytometry analysis. Data are represented as the mean ± S.E.M. (n = 14-16 mice per group). Statistical analysis was performed by Brown-Forsythe and Welch ANOVA test followed by their respective post-hoc test. * *P* < 0.05, ** *P* < 0.01 and *** *P* < 0.001 *vs*. 2 m/o; ^#^ *P* < 0.05 and ^##^ *P* < 0.01 *vs*. 14 m/o.

Since aging is associated with changes in the balance of Cd8^+^ and Cd4^+^ T lymphocyte populations, we evaluated the impact of the chronic treatment with an infratherapeutic dose of APAP in the immune signature of the liver by isolating several populations of non-parenchymal cells (NPCs) followed by flow cytometry analysis. As depicted in Figure 4A, Cd8^+^ T cells were increased in livers from 14 m/o mice in parallel with a reduction in Cd4^+^ T cells. Importantly, both effects were completely prevented by APAP treatment. The decrease in Cd8^+^ T cells in 14 m/o mice receiving APAP was also assessed by immunofluorescence of liver sections (Supplementary Figure 3).

**Figure 5:**
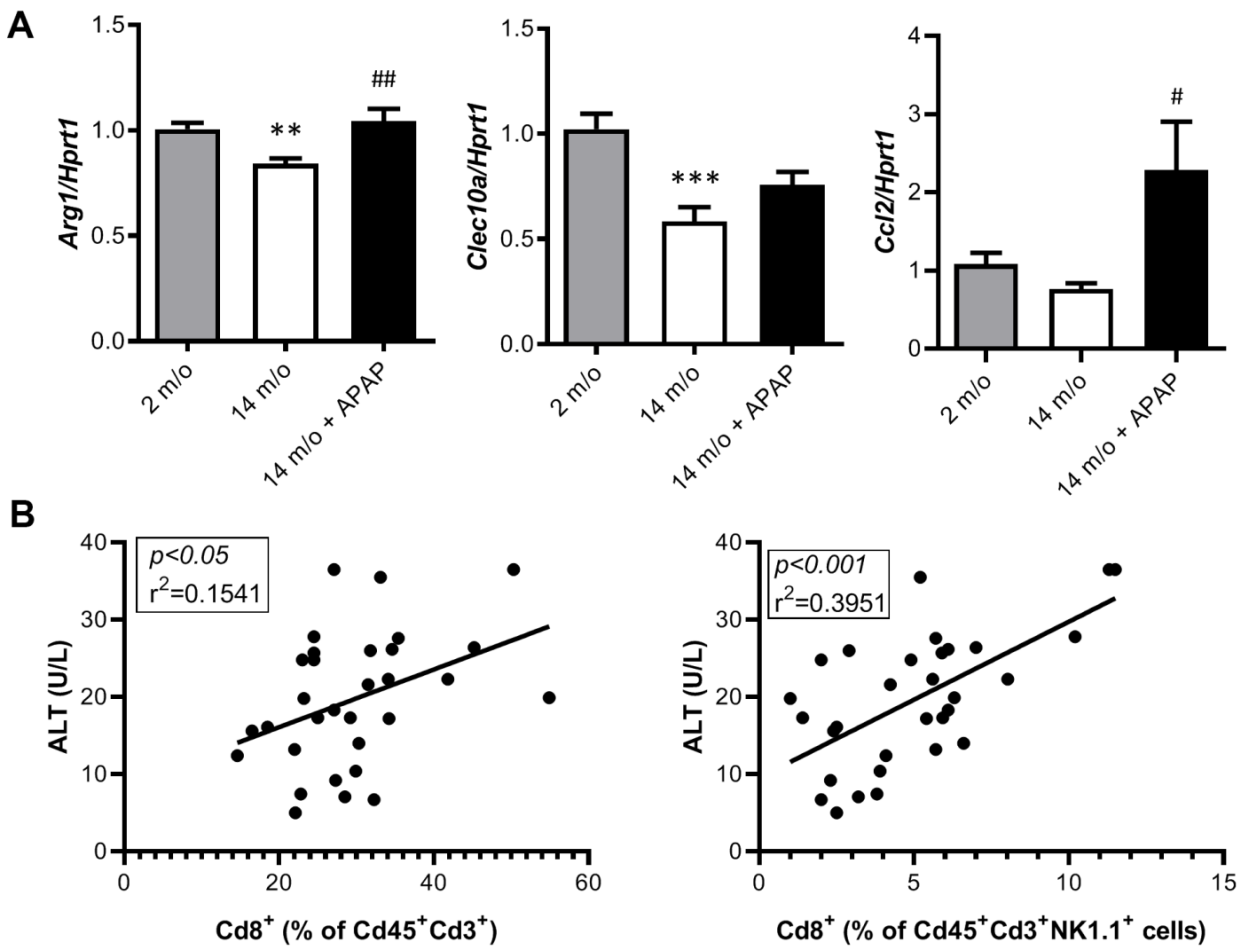
**Analysis of markers of the M2 anti-inflammatory response and correlation of Cd8^+^ T cells with ALT.** (**A**) mRNA expression of *Arg1*, *Clec10a* and *Ccl2* in livers from 2 m/o, 14 m/o and 14 m/o + APAP mice, analyzed by q-PCR. Values were normalized against *Hprt1* mRNA and expressed as fold of change *vs*. 2 m/o. (**B**) Linear regression between plasma ALT activity levels and the 2 populations of Cd8^+^ cells from 2 m/o, 14 m/o, and 14 m/o + APAP mice. Data are expressed as the mean ± S.E.M. (n = 6-16 (**A**), 9-12 (**B**) mice per group). Statistical analysis was performed by Kruskal-Wallis or one-way ANOVA test followed by their respective post-hoc test. ** *P* < 0.01 and *** *P* < 0.001 *vs*. 2 m/o; ^#^ *P* < 0.05 and ^##^ *P* < 0.01 *vs*. 14 m/o.

As reported by Taniguchi and co-workers [11], the NKT system plays a key role in adaptive and innate immune responses. Moreover, the role of the liver as reservoir of NKT cells has been described [12, 13]. Taking this into account, the analysis of Cd8^+^ and Cd4^+^ populations gated from NKT^+^ cells revealed similar pattern than that of Cd3^+^ lymphocytes (Figure 4B). According with these results, we also found a decrease in *Arg1* (encoding Arginase 1), *Clec10a* (encoding MGL1) and *Ccl2* (encoding MCP1) mRNA levels, all markers of the M2 anti-inflammatory response, in 14 m/o mice that were reversed by APAP treatment (Figure 5A). Recently, Haas and co-workers have defined circulating Cd8^+^ T cells as good predictors of NAFLD progression [14]. Interestingly, our results showed a positive correlation between levels of transaminases and the amount of Cd8^+^ cells from both Cd3^+^ and NKT populations (Figure 5B). Moreover, we observed an increase in resident recruited monocytes (Cd11b^+^Ccr2^+^Ly6c^-^) in 14 m/o livers which was reversed by APAP (Supplementary Figure 4A), without changes in other inflammatory cell populations (Supplementary Figure 4B–4E). Immunostaining revealed that the total population of monocytes positive for Ly6c increased in the liver during aging in either untreated or APAP-treated mice also confirmed by flow cytometry (Supplementary Figure 4F). These results suggest that the balance between Cd4/Cd8 T lymphocyte populations plays a more relevant role in the APAP-mediated protective effect rather than monocyte inflammatory cells.

**Figure 6:**
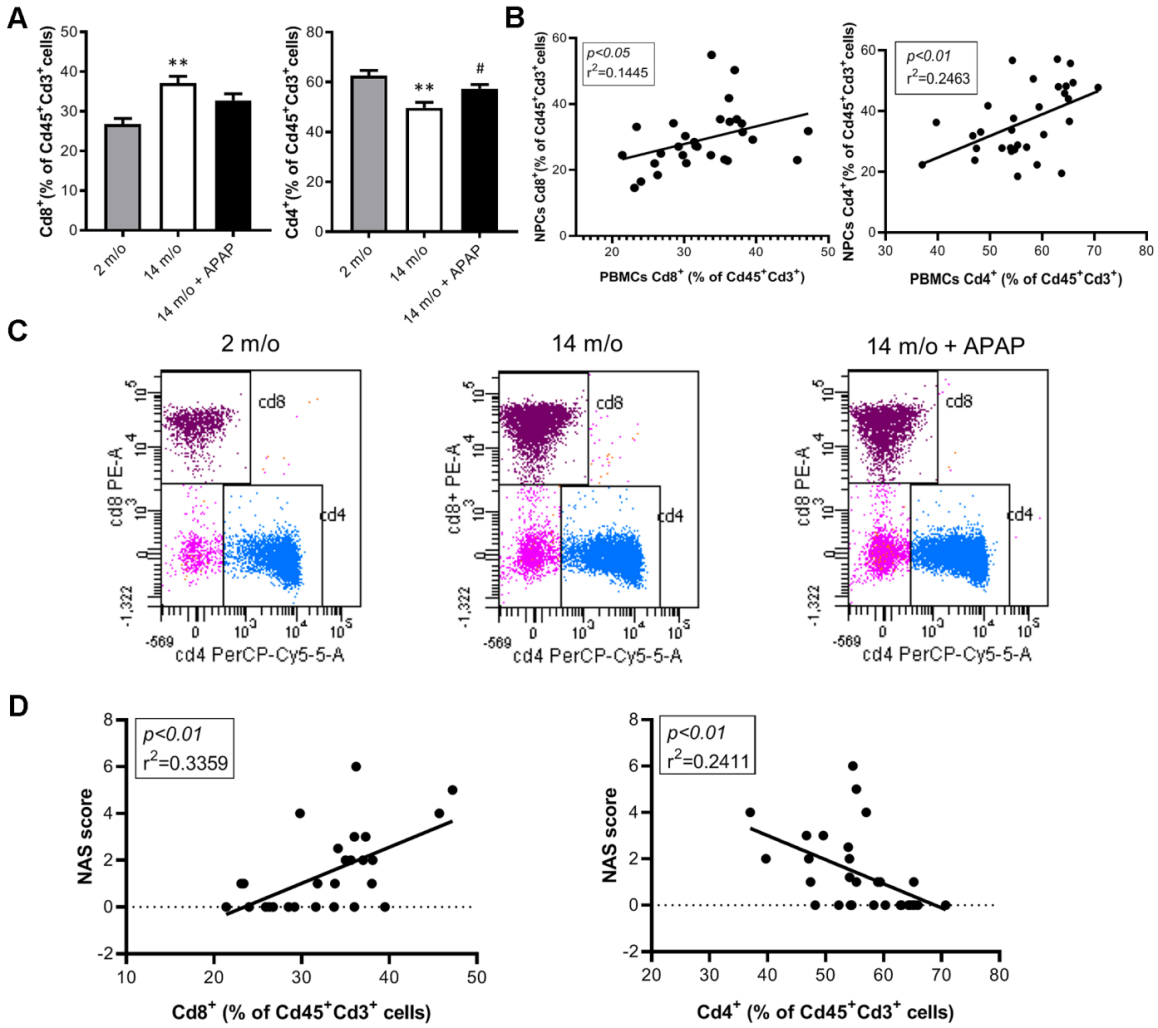
**Analysis of lymphoid populations in PBMCs from 2 m/o, 14 m/o and 14 m/o + APAP mice by flow cytometry.** (**A**) Percentage of Cd8^+^ (left) and Cd4^+^ (right) from Cd45^+^Cd3^+^ cells. (**B**) Linear regression between populations in NPCs and PBMCs from 2 m/o, 14 m/o and 14 m/o + APAP mice. (**C**) Representative images of flow cytometry analysis. (**D**) Linear regression between Cd8^+^ cells (left) and Cd4^+^ cells (right) in PBMCs and the NAS score from 2 m/o, 14 m/o and 14 m/o + APAP mice. Data are represented as the mean ± S.E.M. (n = 7-11 mice per group). Statistical analysis was performed by Brown-Forsythe and Welch ANOVA test followed by their respective post-hoc test. ** *P* < 0.01 *vs*. 2 m/o; ^#^ *P* < 0.05 *vs*. 14 m/o.

**Figure 7:**
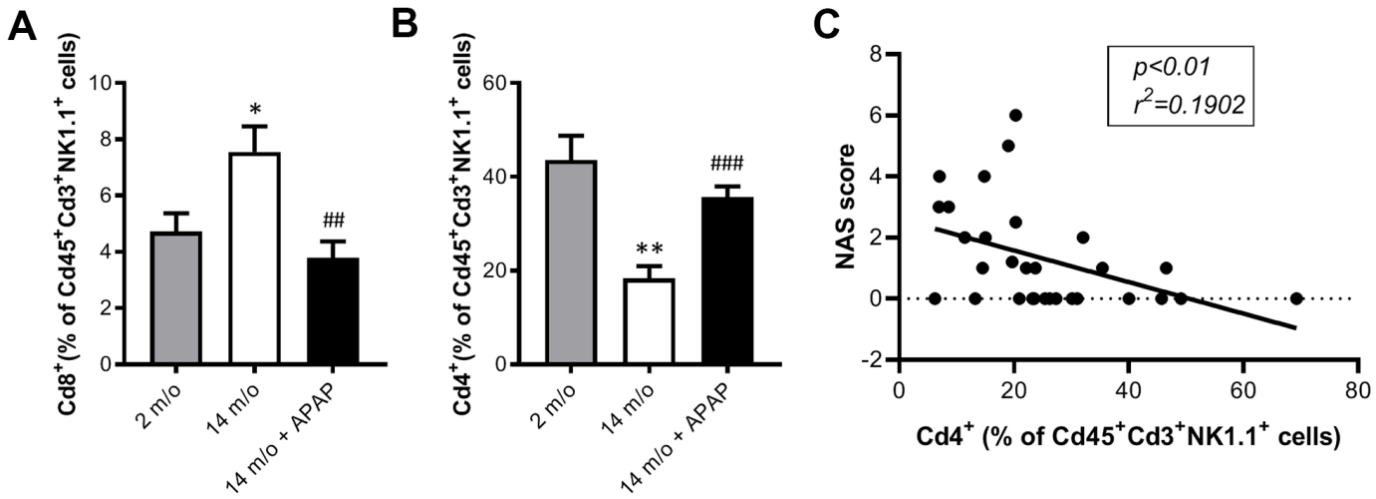
**Analysis of NKT subpopulations in PBMCs from 2 m/o, 14 m/o and 14 m/o + APAP mice by flow cytometry.** (**A**) Percentage of Cd8^+^ cells from Cd45^+^Cd3^+^NK1.1^+^ cells. (**B**) Percentage of Cd4^+^ cells from Cd45^+^Cd3^+^NK1.1^+^ cells. (**C**) Linear regression between NAS score and Cd4^+^ cells from Cd45^+^Cd3^+^NK1.1^+^ cells in PBMCs. Data are represented as the mean ± S.E.M. (n = 9-14 mice per group). Statistical analysis was performed by Brown-Forsythe and Welch ANOVA test followed by their respective post-hoc test. * *P* < 0.05 and ** *P* < 0.01 *vs*. 2 m/o; ^##^ *P* < 0.01 and ^###^ *P* < 0.001 *vs*. 14 m/o.

The absence of validated circulating biomarkers for the evolution of chronic liver diseases is one of the main problems that limit the correct diagnosis of these pathologies. In this context, we analyzed the inflammatory responses in PBMCs of the experimental groups of mice by flow cytometry. In agreement with the data from NPCs, we did not find changes in macrophages and total lymphocytes in PBMCs neither by age or treatment (Supplementary Figure 5A, 5C). Although in NPCs the neutrophils population did not change between groups (Supplementary Figure 4D), in PBMCs this population was increased in 14 m/o mice in comparison with 2 m/o young mice and this effect was not observed in the 14 m/o + APAP group (Supplementary Figure 5B). Importantly, we found a similar pattern in the changes of Cd8^+^ and Cd4^+^ T populations (from Cd3^+^) in NPCs and PBMCs (Figure 6A, 6C) and, interestingly, they were positively correlated (Figure 6B). These results were also confirmed in Cd8^+^ and Cd4^+^ gated from NKT cells of PBMCs (Figure 7A, 7B).

To corroborate the role of circulating Cd8^+^ cells as a potential biomarker of liver disease we analyzed the correlation of this population with the NAS score in our experimental model. The results shown in Figure 6D confirmed a significant positive regression between Cd8^+^ T cells (from Cd3^+^) and the NAS score.

Moreover, Cd4^+^ cells (from Cd3^+^ and also Cd3^+^NK1.1^+^) were inversely correlated with the NAS score (Figures 6D, 7C).

## DISCUSSION

To date, age-related changes in the metabolism after APAP exposure are poorly understood. In fact, very few studies have investigated the effect of chronic treatment with APAP in hepatotoxicity, since most of the work has been focused in identifying the molecular mechanisms responsible of fulminant liver failure upon APAP intoxication [32–34]. Herein, the objective of this study was to evaluate the impact of chronic APAP administration in middle-aged mice in metabolic-related parameters with a major focus in the inflammatory responses of the liver. For this purpose, we administered the infratherapeutic APAP dose of 57.1 g/kg/day added to the drinking water to mice during 8 months since our previous study showed APAP-protein adducts, but not hepatotoxicity in this intervention [35]. The starting time-period (6 months of age) of APAP administration was chosen to delineate a stage of middle age, time at which metabolic risks arise [36, 37].

To date, it is recognized that the genetic background of the preclinical animal models can impact on their metabolic phenotype [38–40]. In our previous studies [35, 41] mice on the C57BL/6J x 129Sv/J (B6;129Sv) genetic background were investigated at different ages, while mice on B6D2JRcc/Hsd genetic background, highly susceptible to diet-induced metabolic and liver damage [22, 23], were used in the present study. In fact, differences in body weight among the two genotypes became more evident with age indicating a background-specific susceptibility to weight-related metabolic alterations through aging. These results suggest that APAP chronically administered in a more obesogenic background could mask its effects on metabolic alterations. However, differences in APAP metabolism including expression levels of CYP2E1 or induction of protective factors such as FGF15/19 [42, 43] could also account for these differences.

Specific age-related changes in the liver have been previously reported including increased hepatocyte size, binucleated cells, augmented lobular inflammation, and reduction in mitochondrial number [2, 44]. Overall, these changes may significantly affect liver morphology, physiology and oxidative capacity. Herein we show that whereas aging led to a significant increase in steatosis and inflammation in the liver, it only modestly elevated transaminases and LDH, pointing to mild alterations. From a therapeutic perspective, reversion or prevention of age-related liver damage might improve health and lifespan in the elderly [45, 46]. In this regard, the above-mentioned age-related effects in the liver were prevented by chronic treatment of B6D2JRcc/Hsd mice with an infratherapeutic dose of APAP, suggesting a protective effect of this intervention independently of body weight gain or glucose metabolism.

Oxidative stress may be one of the causal factors underlying metabolic dysfunction during aging due to a decline of the antioxidant defenses [26, 47–49]. Accordingly, our results illustrated reduced HO-1 and MnSOD protein levels in 14 m/o mice. Also, in line with previous studies [50–52], those mice showed an increase in NQO1 protein and *Nqo1* mRNA suggesting a protective mechanism to counteract the aging process. By contrast, other studies reported increased levels of HO-1 in the liver during aging [53, 54]. More importantly, chronic treatment with APAP at infratherapeutic dosage enhanced the antioxidant response of the liver by increasing HO-1, MnSOD and NQO1 expression, thereby supporting the protective effect of this intervention. Notably, HO-1 was also found elevated in rat livers between 12 and 36 h after exposure to an APAP overdose [55, 56] and, in this line, HO-1 induction is well recognized as a cytoprotective response against liver injury by suppressing oxidative stress and inflammation [57, 58]. Also, reduction of MnSOD activity aggravates APAP-induced liver injury and mitochondrial toxicity in mice [59, 60]. On the other hand, Moffit et al*.* reported that after an APAP overdose NQO1 enzymatically reduces NAPQI to be converted in APAP, partially decreasing the deleterious effect of this reactive metabolite [61]. Likewise, Mach et al*.* demonstrated that old rats acutely treated with an APAP overdose were more protected against hepatotoxicity than young animals due to reduced formation of NAPQI [51]. Taking this into account, we hypothesize that age-related increased NQO1 likely contributes to the protective effect of APAP, since both aging and APAP cooperated in preventing the accumulation of NAPQI. Of note, our results did not show translocation of NRF2 to the nucleus either with age or APAP, suggesting that alternative mechanisms are required to upregulate the antioxidant enzymes. In fact, HO-1, NQO1 and MnSOD expression is also regulated by NRF2-independent mechanisms [62–64]. Importantly, we also evidenced that age-induced LPO and DHE-derived fluorescence are counteracted after chronic APAP treatment, an effect likely mediated by these antioxidant enzymes. It is important to highlight that mild oxidative stress has been proposed as a positive hormetic event since low protein and lipid oxidative damage may have prophylactic effects over several diseases associated to aging [65]. In this context, “hormesis” is defined as a process in which exposure to a low dose of a chemical agent or environmental factor that is damaging at higher doses induces an adaptive beneficial effect on the cell or organism [66, 67]. Therefore, the chronic infratherapeutic APAP treatment performed in our study could be an anti-aging hormetic mechanism by promoting adaptive biological responses to counteract deleterious liver alterations induced by aging.

Previous publications pointed ROS or aging-induced activation of MAPK family proteins in various tissues including brain, lung, muscle, and liver [68–70]. Thus, MAPK signaling might be increased as indicative of chronic stress through life, even in the absence of a toxic challenge. In this regard, phosphorylation levels of ERK1/2 and p38 MAPK were significantly increased in livers from 14 m/o mice, an effect markedly attenuated to levels found in young mice after chronic treatment with APAP at an infratherapeutic dosage in parallel with a reduction in ROS levels. Thus, targeting ERK1/2 and p38 MAPK activities may be crucial in delaying age-related liver alterations.

During aging, adaptive immunity declines, whereas innate immunity seems to be activated which induces a characteristic proinflammatory profile regulated by NF-κB [71]. Contrary to our findings after an acute toxic APAP exposure [21], activation of the NF-κB signaling pathway was not detected upon chronic exposure to infratherapeutic doses of the drug. These results are consistent with the fact that APAP at this infratherapeutic dosage does not cause liver damage, but rather favors a protective effect.

Previous studies have shown that NPCs play a role in physiological liver functions as well as in acute liver damage, such as drug-induced liver injury (DILI), hepatitis, as well as in acute inflammation, and in chronic liver diseases, such as liver fibrosis and cirrhosis [72]. In this regard and due to the difficulties in evaluating inflammatory processes in the whole liver, we analyzed in detail the NPCs populations by flow cytometry. It is well-known that aging is associated with immunosenescence, a complex immune remodeling process that affects particularly Cd3^+^Cd8^+^ T cells [73]. In agreement, Cd3^+^Cd8^+^ T population increased in livers from 14 m/o mice revealing for the first time liver-specific age-dependent immunomarkers with a similar profile recently found in mice with NAFLD [14, 74]. In fact, in livers from 14 m/o mice both steatosis and inflammation NAS scores resemble those of mice with fatty liver reported by our group [75, 76]. More importantly, a recent study in humans with different stages of NAFLD revealed a linear correlation between steatosis, inflammation and global NAS scores with hepatic Cd8^+^ T cells [14], also in agreement with our data. Notably, this increase in Cd8^+^ T cells found in human livers with NAFLD was more evident in non-diabetic patients, also coincident with the absence of alterations in systemic glucose homeostasis in our mice model. It is noteworthy to mention that the decrease of Cd3^+^Cd4^+^ T cells is also an immune signature of 14 m/o mice that correlates with increased LPO and DHE staining together with a drop of antioxidant defenses (*i.e.* HO-1 and MnSOD). Again, our results are in agreement with data reported by Ma et al*.* in a mouse model of NAFLD showing a reduction of the Cd3^+^Cd4^+^ population as a result of the dysregulation of lipid metabolism and the increase in mitochondrial ROS [77]. Regarding Cd3^+^NK1.1^+^Cd8^+^ population, the increase found in livers from 14 m/o mice agrees with the pattern previously described by our group [78] and others [79] in mice with non-alcoholic steatohepatitis (NASH) and also in human NASH patients [80]. Besides NAFLD, to the best of our knowledge the increase in this circulating cell population with aging has been reported, but not in the liver, highlighting the relevance of our findings regarding the immune signature of the liver thorough age. Finally, we found higher recruited myeloid cells (Cd11b^+^Ccr2^+^Ly6c^-^) in 14 m/o mice livers as also reported in obese mice [81]. Importantly, our data revealed that chronic treatment with APAP at an infratherapeutic dose protects against the imbalance in liver immune NPCs thorough life. These data were corroborated by increased *Arg1* and *Ccl2* mRNA levels, a readout of M2 stage. The benefit of this chronic treatment on liver inflammation might be explained by the above mentioned effect of APAP in enhancing antioxidant defenses, thereby limiting hepatic inflammation. In fact, as discussed above, the drop of Cd3^+^Cd4^+^ T cells during NAFLD was attributed to mitochondrial ROS [77]. Thus, it is reasonable to suggest that this also occurs in the liver during aging. Similar direct relationship (oxidative stress and inflammation) can be extended to the elevations of cytotoxic lymphocytes and NKTs found in 14 m/o mice that did not receive APAP and manifested increased LPO and DHE staining. More interestingly, Cd8^+^ T cells of the liver, including both total and the specific NK1.1^+^Cd3^+^ population, correlated with transaminase levels, pointing to this population as an indicative of liver inflammatory damage with potential diagnostic value. Thus, this experimental approach is likely valuable as predictor of unhealthy liver through life, particularly in middle age individuals at risk to develop metabolic disturbances.

Due to the limitations in obtaining liver samples for diagnostic purposes, and based on the study of Haas et al*.* correlating blood Cd8^+^ T lymphocytes with the NAS score in NASH patients [14], we analyzed Cd8^+^ and Cd4^+^ lymphocytes in PBMCs and found similar profile than hepatic NPCs in all experimental groups. Of relevance, these inflammatory populations in PBMCs linearly correlated with the NAS score of the mice. Moreover, hepatic and blood lymphocytes correlated with a broad range of NAS score, pointing to PBMCs as potential biomarkers for diagnosis and monitoring age-associated hepatic chronic inflammation frequently associated to metabolic diseases.

Collectively, our data suggest that chronic administration of an infratherapeutic dose of APAP exerts hepatic protection against liver alterations during aging by maintaining the balance of the redox status and preventing immunosenescence that ultimately favors healthy aging. We also described a correlation between liver damage and an immune signature in liver and PBMCs pointing PBMCs as potential biomarkers of liver alterations during aging.

## MATERIALS AND METHODS

### Chemicals

The antibodies were obtained from Merck-Sigma Aldrich (St. Louis, MO, USA), Cell Signaling Technology (Danvers, MA, USA), Santa Cruz Biotechnology (Dallas, TX, USA), Calbiochem/Merck Millipore (Billerica, MA, USA), and Enzo Life Sciences (Farmingdale, NY, USA). Reagents used for electrophoresis and Western Blot (WB) were purchased from Bio-Rad (Hercules, CA, USA).

### Animal experimentation

Two and 14 months-old male mice (hereafter referred as 2 m/o and 14 m/o, respectively) with mixed genetic background B6D2JRcc/Hsd were used throughout this study. Mice were kept in cycles of 12 h of light/dark in temperature (22° C) and humidity-controlled rooms, fed standard chow diet *ad libitum* and with free access to drinking water. Male animals at the age of 6 months were randomly distributed into control and APAP experimental groups. APAP treated-mice were given an infratherapeutic dose around 57.1 mg/kg/day (Merck-Sigma Aldrich) (equivalent to 324.08 mg/day for a 70 kg adult human) [82] mixed in the drinking water for 8 months (hereafter referred as 14 m/o + APAP). Control (age matched 14 m/o and 2 m/o) mice received regular drinking water. During the treatment, body weight and water consumption were measured weekly. To achieve the desired dosage levels, the concentration of APAP in the drinking water was adjusted weekly according to the body weight and water consumption of each group. At the end of the treatment mice were sacrificed. Blood was collected by decapitating the animals and processed for biochemical parameters. Liver was immediately frozen in liquid nitrogen and stored at -80° C for subsequent molecular analysis, or fixed with 4 % paraformaldehyde (PFA) for further histological analysis. All animal experimentation was carried out in accordance with the recommendations of the Federation of European Laboratory Animal Science Associations (FELASA) on health surveillance, the European Community Law (2010/63/UE) and the Spanish Law (RD 53/2013), with the approval of the Ethics Committee of the Bioethics Commission of the CSIC, Spain.

### Histopathology assessment

Paraffin-embedded liver biopsy sections (5 μm) were stained with hematoxylin and eosin (H&E) at the CNB Histology Facility (Madrid, Spain) and evaluated by a single blinded hepatopathologist (C. G-M). NAFLD activity score (NAS score) was determined by using the histological evaluation system for animal models previously validated [83]. The degrees of steatosis of hepatocytes were divided into: 0, < 5 %; 1, 5-33 %; 2, > 33-66 %; and 3, > 66 %. The lobular inflammation was divided into: 0, without foci (< 0.5 foci); 1, 0.5-1 foci; 2, 1-2 foci; and 3, > 2 foci. The NAS score was calculated based on the set of grades of steatosis and lobular inflammation.

### Oil Red O staining

For Oil Red O staining, pieces of liver were fixed O/N in 4 % PFA and cryoprotected in subsequent gradients of 15 % and 30 % sucrose in PBS at 4° C. After, samples were quickly frozen in Tissue-Tec OCT (Miles) and sectioned at 5 μm using a Leica CM3050 S cryostat. Tissue slices were fixed in ice-cold 10 % formaldehyde (47630, Fluka) for 10 min and washed twice in distilled water. Then, samples were fixed in 1,2 propyleneglycol (82280, Merck-Sigma Aldrich) for 10 min at room temperature (RT). Slices were stained in 0.7 % Oil Red O solution (O0625, Merck-Sigma Aldrich) shaking for 7 min. After washing twice in 87 % glycerol (A3739, Panreac AppliChem), sections were counterstained with hematoxylin (H3136, Merck-Sigma Aldrich) solution for 5 s, rinsed again with tap water and mounted in Aquatex (108572, Merck- Millipore).

### Isolation of hepatic non-parenchymal cells (NPCs)

NPCs were isolated as described previously [78]. Briefly, livers were collected and washed with warm PBS. Immediately, they were transferred to HBSS (Hank’s Balanced Salt solution; Gibco, Life Technologies, Carlsbad, CA, USA) at RT, disintegrated and filtered through 100 μm cell strainers. Then, homogenates were centrifuged at 500 x g for 5 min at RT and cell pellets were resuspended in a 36 % Percoll solution (GE Healthcare Bio-Sciences AB, Uppsala, Sweden) containing 100 UI/mL of heparin (HIBOR 5000 UI, ROVI Contract manufacturing, Madrid, Spain). After centrifugation at 800 x g without brake for 20 min at RT, supernatants were discarded and then erythrocytes were removed from cell pellets by using a red blood cell lysis buffer (150 mM NH_4_Cl, 10 mM KHCO_3_, 0.1 mM EDTA pH 7.3). The resulting cell pellets were washed with cold HBSS, centrifuged at 500 x g for 5 min at 4° C and, finally, NPCs were resuspended in cold HBSS for further analysis.

### Flow cytometry

Isolated NPCs were incubated with the following antibodies: Cd45-FITC (1/200) (rat IgG, Beckman, Brea, CA, USA), Ly6g-PE (1/100) (rat IgG2ak Pharmigen, San Jose, CA), F4/80-APC (1/25) (rat IgG2ak, eBioscience, San Diego, CA), Cd11b-Pcy7 (1/100) (rat IgG2bk, eBioscience), Cd3-Pcy7 (1/100) (hamster IgG, BioLegend, San Diego, CA), NK1.1-APC (1/100) (mouse IgG2ak, Pharmigen), Cd8-PE (1/50) (rat IgMk, Pharmingen), Cd4-PERCy5.5 (1/100) (rat IgG2bK, BioLegend), Ly6c-FITC (1/100) (rat IgG2bK, BioLegend) and Ccr2 (1/200) (rat IgG2bK, BioLegend); or their corresponding isotype controls for 20 min at RT protected from the light. After washing steps, NPCs were resuspended with PBS. Flow cytometry data were acquired with a FACSCanto II and data analysis was performed using Cytomics FC500 with the CXP program. The results were referred to the % Cd45 positive cells, according to each combination.

### Flow cytometry analysis of peripheral blood mononuclear cells (PBMCs)

Peripheral blood samples were obtained by decapitating the animals and collected with heparin (1000 U/ml, Hospira, ROVI Contract manufacturing, Madrid, Spain). Fifty μl of blood were incubated with the antibodies detailed in the NPCs section for 20 min at RT and protected from the light. Then, erythrocytes were removed by using the red blood cell lysis buffer mentioned above. After 10 min, 2.5 ml of FACS buffer (PBS-0.5 % BSA) were added and samples were centrifuged at 500 x g for 5 min at RT. Cell pellets were washed with FACS buffer and centrifuged again. Cells were resuspended in 200 μl of FACS buffer and then analyzed by flow cytometry after filtration. Flow cytometry data were acquired with a FACSCanto II and data analysis was performed using Cytomics FC500 with the CXP program.

### Alanine transaminase (ALT), aspartate aminotransferase (AST) and lactate dehydrogenase (LDH) assays

The levels of ALT, AST and LDH were determined in plasma by using Reflotron strips (Roche Diagnostics, Barcelona, Spain) for ALT or a specific colorimetric kit according to the manufacturer’s instructions (BioSystems, Barcelona, Spain) for AST and LDH.

### Measurement of intracellular redox state

Lipid peroxidation (LPO) levels were determined as an indirect measure of the production of reactive oxygen species (ROS). The reaction of thiobarbituric acid (TBA) was used according to Ohkawa et al. to quantify *ex vivo* the amount of aldehyde products generated by LPO in liver tissue [84]. Liver extracts (25 mg) were homogenized with 300 μl of 0.15 M KCl and then, centrifuged at 1000 x g for 5 min at RT. Supernatants were collected and 50 μl were incubated with 0.26 % TBA pH 3-3.5, 0.4 % SDS and 7.5 % acetic acid for 60 min at 95° C, and after that, centrifuged at 1000 x g for 5 min at RT. A pink chromophore was produced in samples in direct relation to the amount of peroxidized products. The absorbance of the supernatant was measured in a Synergy HT plate reader (BioTek, Oviglia TO, Italy) at 532 nm. The amount of TBA reactive species (TBARS) (mostly malondialdehyde, MDA) was calculated by interpolation of values in a constructed MDA standard curve with 1,1,3,3-tetrametoxypropane (TMP). Results were expressed as mmol MDA per mg of protein.

### Dihydroethidium (DHE) staining

*In situ* staining of O_2_^•−^ was performed using frozen mice liver biopsy sections as described [85]. Tissue was fixed in 4 % PFA during 24 h, after that it was embebed in sucrose gradient and then frozen in Tissue-Tek OCT and stored at −70° C until use. Frozen liver tissue sections (20–30 μm) were preincubated with a superoxide dismutase mimetic MnTBAP (10 μM, Sigma Aldrich) during 1 h at RT. For *in situ* imaging of O_2_•−, slides were incubated for 30 min in a humidified chamber at 37° C with 5 μM DHE (Molecular Probes, Inc, USA). Images were obtained with a Zeiss 510 laser scanning confocal microscope and fluorescence intensity of liver sections (5 sections per mice, n=5 mice/group) was analyzed with ImageJ software (National Institutes of Health).

### Fluorescent immunohistochemistry

Livers were dissected and fixed in 4 % PFA O/N at 4° C. Livers were transferred to 15 % sucrose in PBS and 30 % sucrose in PBS at 4° C until tissue sunk and, then embedded in Tissue-tek OCT. For immunofluorescence, 5 μm OCT tissue cryosections were stained with anti-Cd8 antibody (#550281, BD), incubated with donkey anti-rat Alexa Fluor 595 secondary antibody (#A21209, Thermofisher) and counterstained with DAPI. Random field (6 per mice) were collected using a Nikon Eclipse 90i microscope and positively stained cells were quantified using ImageJ Software (NIH).

### Chromogenic immunohistochemistry

Livers were dissected and fixed in 4 % PFA O/N at 4° C and embedded in paraffin. Sections of 5 μm thickness were cut and stained with anti-Ly6c antibody (provided by A. Castrillo, CSIC, Spain), incubated with biotin-conjugated rabbit anti-rat secondary antibody (#BA-4000, Vector) and counterstained with Harris Hematoxylin. Random fields (6 per mice) were obtained using a Zeiss AxioPhot microscope and positively stained cells were quantified using ImageJ Software (NIH).

### RNA extraction and quantitative polymerase chain reaction (qPCR) analysis

Total RNA from liver samples was extracted using TRIzol reagent (Thermofisher Scientific, MA, USA), and treated with DNAse for 10 min according to the manufacturer’s instructions. Equal amounts (250 ng) of RNA were reverse transcribed using the High Capacity cDNA Reverse Transcription Kit (Thermofisher Scientific) as follows: 25° C, 10 min; 37° C, 2 h; 85° C, 5 min. Quantitative PCR (qPCR) was performed with 50 ng of cDNA mixed with Power SYBR Green Master Mix (Thermofisher Scientific) for primers (Table 1) or with Taqman Universal Master Mix II (Thermofisher Scientific) for Taqman probes (Table 1). Amplification was conducted in a 7900HT Fast-Real Time PCR System (Life Technologies). PCR cycles proceeded as follows: initial denaturation for 10 min at 95° C and then 40 cycles of 15 s at 95° C and 1 min at 60° C. For qPCR using SYBR Green Master Mix, the dissociation curve was performed as follows: 15 s at 95° C; 15 s at 60° C; and 15 s at 95° C, in order to confirm the specificity of the PCR products. Data analysis is based on the ΔΔCt method with normalization of the raw data to the housekeeping gene Hypoxanthine phosphoribosyl transferase 1 (*Hprt1*), as described in the manufacturer’s manual (Life Technologies). Each sample was analyzed in duplicate.

**Table 1:** List of primers used for qPCR analysis.

**Gene**	**Taqman reference/** **Primer sequence 5′-> 3′**
*Arg1*	Mm00475988_m1
*Hprt1*	Mm00446968_m1
*Clec10a* (encoding MGL1)	F: 5’-TGAGAAAGGCTTTAAGAACTGGG-3’ R: 5’-GACCACCTGTAGTGATGTGGG-3’
*Hmox1* (encoding HO-1)	F: 5’-AGGCTAAGACCGCCTTCCT-3’ R: 5’-TGTGTTCCTCTGTCAGCATCA-3’
*Nfe2l2* (encoding NRF2)	F: 5’-TAGATGACCATGAGTCGCTTGC-3’ R: 5’-GCCAAACTTGCTCCATGTCC-3’
*Sod2* (encoding MnSOD)	F: 5’-TCAGTGCTCACTCGTGTCAT-3’ R: 5’-ACACGATAGGTTTGGGCATA-3’
*Nqo1*	F: 5’-GTCCATTCCAGCTGACAACCA-3’ R: 5’-TTGCCCTGAGGCTCCTAATC-3’
*Ccl2* (encoding MCP1)	F: 5’- AAAAACCTGGATCGGAACCAA-3’ R: 5’- CGGGTCAACTTCACATTCAAAG-3’
*Hprt1*	F: 5’-GAGGAGTCCTGTTGATGTTGCCAG-3’ R: 5’-GGCTGGCCTATAGGCTCATAGTGC-3’

### Homogenization and preparation of tissue extracts and western blotting

Liver tissue (30-50 mg) was homogenized in ice-cold lysis buffer containing 0.5 % CHAPS, 10 mM Tris-HCl pH 7.5, 1 mM MgCl_2_, 1 mM EDTA, 10 % glycerol and protease and phosphatase inhibitors (5726, P0044, P8340, Merck-Sigma Aldrich) using the Brinkman PT 10/35 Polytron. Extracts were then incubated for 45 min at 4° C with continuous agitation. Liver extracts were cleared by centrifugation at 13000 x g for 15 min at 4° C. Protein determination was performed by the Bradford dye method, using the Bio-Rad reagent and bovine serum albumin (BSA) (Merck-Sigma Aldrich) as the standard. Absorbance was measured in a Synergy HT plate reader at 595 nm.

Cytosolic and nuclear extracts were isolated from 25 mg of tissue as follows. Livers were disrupted in 0.3 M sucrose solution containing the protease and phosphatase inhibitors mentioned above by using a micro pestle. Homogenates were centrifuged at 1000 x g for 10 min at 4° C to separate the cytosolic fraction (supernatant) and the nuclear fraction (pellet). Supernatants containing the cytosolic fraction were centrifuged 3 times at 27000 x g for 15 min at 4° C, and transferred to a new tube for protein analysis. Pellets containing the nuclear fraction were washed with 0.3 M sucrose solution and centrifuged at 1000 x g for 10 min at 4° C. After three washing steps, pellets were then resuspended in RIPA buffer (Merck-Sigma Aldrich) and incubated for 1 h at 4° C on a rotating mixer. After sonication, cellular debris was removed by centrifugation at 8000 x g for 15 min at 4° C and the supernatants containing the nuclear fraction were collected. Protein determination was performed using the Pierce BCA Protein Assay Kit (Thermofisher Scientific) and a standard curve of BSA. Absorbance was measured in a Synergy HT plate reader at 562 nm.

For protein analysis, protein extracts (30 μg) were boiled for 5 min with Laemmli buffer (200 mM Tris-HCl pH 6.8, 5.2 % SDS, 40 % glycerol, 3 % β-mercaptoethanol, and 0.08 % bromophenol blue) and then separated by 8-12 % SDS-PAGE (80 V-120 V, 2 h). Proteins were transferred to a polyvinylidene fluoride membrane (PVDF) using a semi-dry method (Trans-blot SD SEMI-Dry Electrophoresis Transfer Cell, Bio-Rad; 25 V and 1.5 A, 37 min). Membranes were blocked using 5 % non-fat dried milk in PBS for 1 h at RT and then incubated with primary antibodies in PBS (Table 2) overnight at 4° C. After 3 washes with PBS containing 0.1 % Tween-20, membranes were incubated with the corresponding peroxidase-conjugated secondary antibodies (anti-rabbit, 1/5000, Bio-Rad; anti-goat, 1/50000, Merck-Sigma Aldrich) for 1 h at RT. Immunoreactive bands were visualized using the ECL Western blotting protocol. Blots were normalized by lane charge using antibodies against GAPDH, VINCULIN or LAMIN B, as indicated in each figure. Different exposure times were used for each membrane to ensure the linearity of the intensity of the bands. The densitometric analysis was carried out with the ImageJ program and was expressed in arbitrary units.

**Table 2:** List of primary antibodies used for analysis by WB.

**Detects**	**Commercial manufacturer**	**Reference**
pERK1/2	Cell Signaling	4370P
ERK1/2	Cell Signaling	9102S
pp38	Cell Signaling	9211S
p38	Cell Signaling	9212
HO-1	Merck Millipore	AB1284
MnSOD	Enzo Life Sciences	ADI-SOD-MOF
NQO1	Cell Signaling	3187
VINCULIN	Santa Cruz Biotechnology	sc-73614
GAPDH	Merck-Sigma Aldrich	G9545
NRF2	Kindly provided by Dr. Cuadrado (UAM, Spain)	
p65-NF-κB	Cell Signaling	8242
LAMIN B	Santa Cruz Biotechnology	sc-6217
pIκBα	Cell Signaling	9246
IκBα	Cell Signaling	9242

### Glucose and pyruvate tolerance tests (GTT, PTT)

For glucose tolerance tests, 16 h fasted mice received an intraperitoneal (i.p.) injection of 2 g D-glucose/kg body weight. Glucose concentration in blood was measured with an Accu-Check glucometer (Roche Diagnostics) at 0, 15, 30, 60, 90 and 120 min time points, and the area under the curve (AUC) was calculated. Pyruvate tolerance tests were performed similarly, except that the mice received an i.p. injection of 1.5 g sodium pyruvate/kg body weight.

### Data analysis

Statistical analysis was performed using Kruskal-Wallis, one-way ANOVA or Brown-Forsythe and Welch ANOVA tests, followed by Dunn, Bonferroni or Tamhane T2 post-hoc tests, respectively. No-parametric Mann-Whitney U test was also used. The results were expressed as the mean ± standard error (S.E.M.) of at least four-six animals. To explore the effect to APAP treatment in body weight throughout the treatment, mixed ANOVA of repeated measures was used to analyze the effect on the age, treatment and the age-treatment interaction. The Mauchly sphericity test was used to test the sphericity hypothesis. In the case of rejection, the analyses were based on the Greenhouse-Geisser multivariate test. The analysis was performed with the GraphPad Prism 8 program. The level of significance was defined as *P* < 0.05.

## Supplementary Material

Supplementary Figures
