# Supplementary material for: Chronic treatment with acetaminophen protects against liver aging by targeting inflammation and oxidative stress
Source: Aging (Albany NY). 2021 Mar 29;13(6):7800–27. doi: 10.18632/aging.202884 (PMC8034963; doi:10.18632/aging.202884)
Supplement: Supplementary Figures [file aging-13-202884-s001.pdf]

SUPPLEMENTARY FIGURES

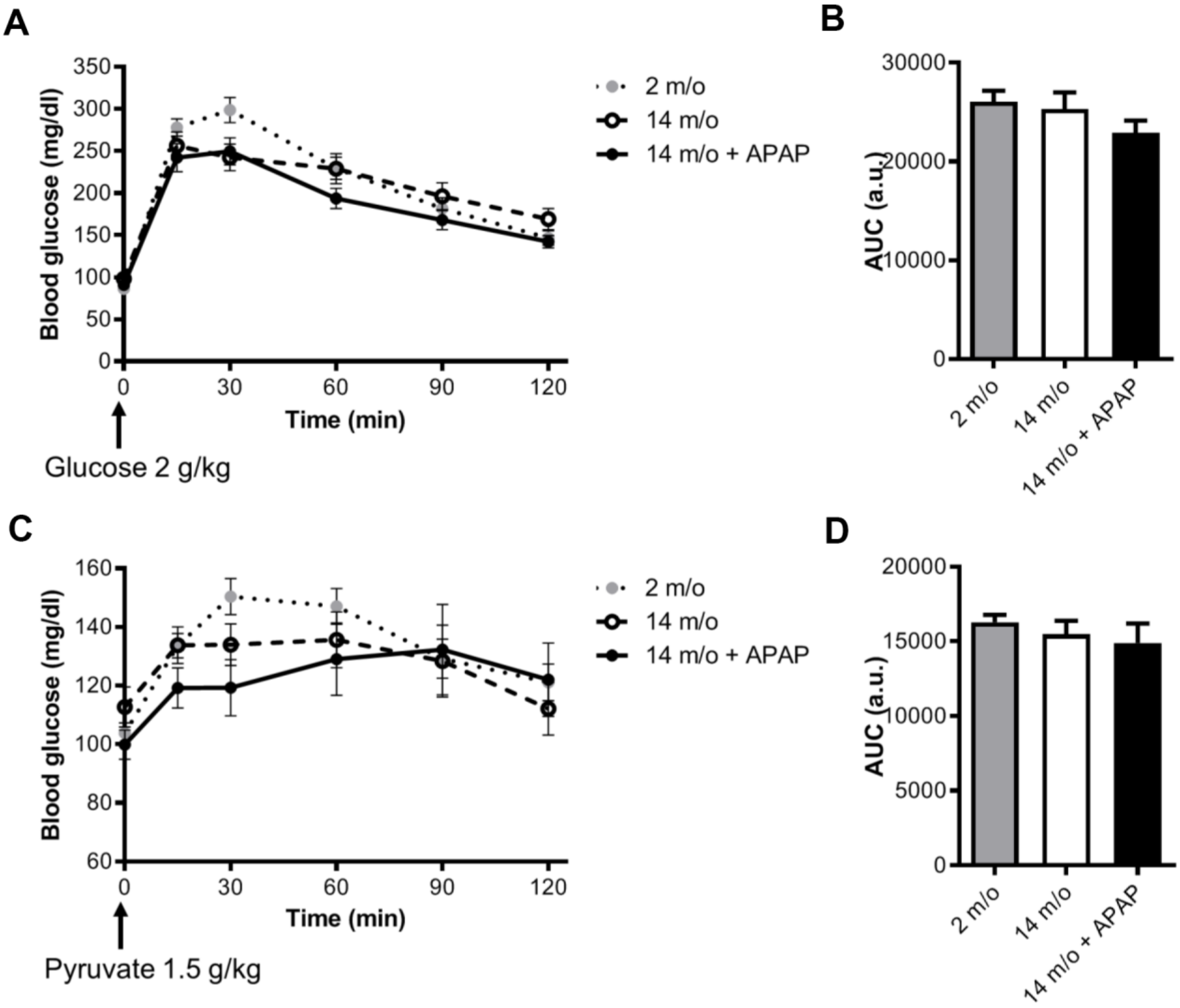

Supplementary Figure 1. Chronic APAP exposure at an infratherapeutic dose did not alter glucose homeostasis in 14 m/o mice. (**A**) GTT from 2 m/o, 14 m/o and 14 m/o + APAP mice. Values showing blood glucose (mg/dl) correspond to mean ± S.E.M. (n = 18-22 mice per group). (**B**) Graph depicts the area under the curve (AUC) from (**A**). (**C**) PTT from 2 m/o, 14 m/o and 14 m/o + APAP mice. Values showing blood glucose (mg/dl) correspond to mean ± S.E.M. (n = 17-22 mice per group). (**D**) Graph depicts the area under the curve (AUC) from (**C**).

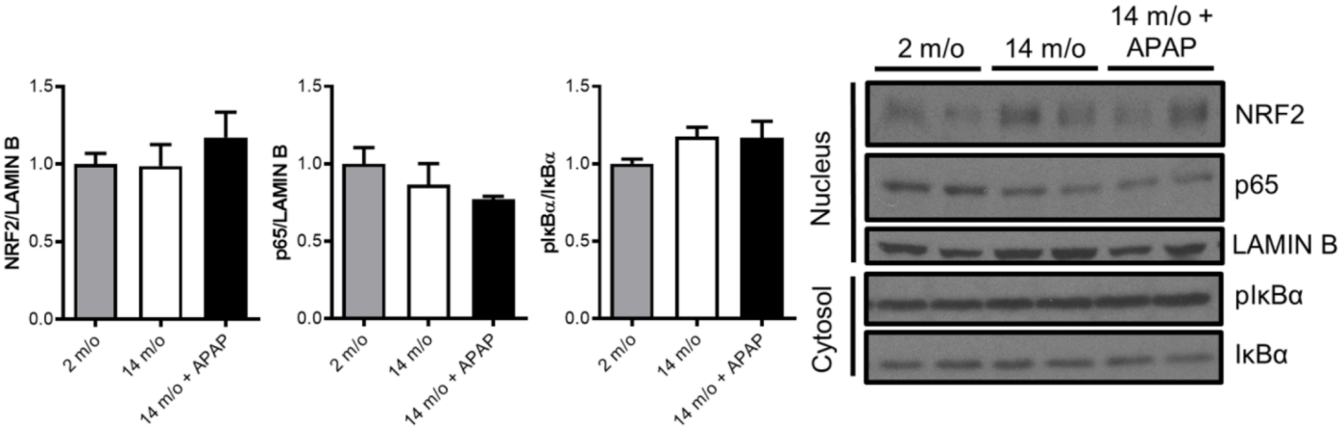

Supplementary Figure 2. Impact of chronic APAP treatment in NRF2 and NF-κB signaling pathways. Representative immunoblots showing nuclear NRF2 and p65-NF-κB level using LAMIN B as a loading control, and cytosolic p-IκBα *vs*. total IκBα. Graphs depict densitometric quantifications of the indicated protein levels. Values are mean ± S.E.M. (n = 6-8 mice per group) (see original western blot in Supplementary Figure 8). Statistical analysis was performed by one-way ANOVA or Brown-Forsythe and Welch ANOVA test followed by their respective post-hoc test.

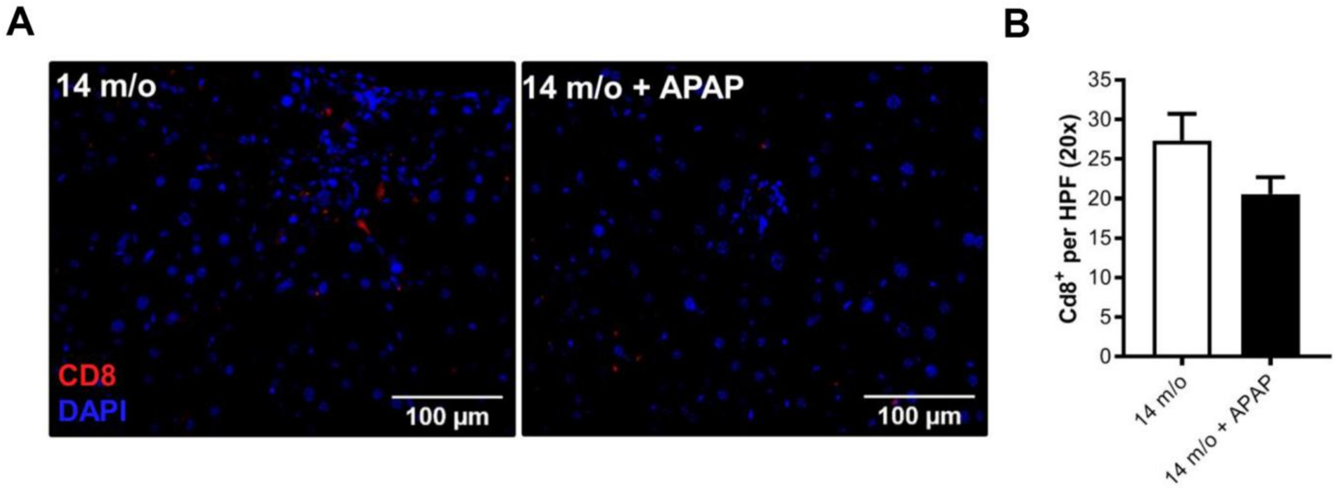

Supplementary Figure 3. Quantification of Cd8 positive cells in liver sections from 14 m/o and 14 m/o + APAP mice by immunofluorescence. (**A**) Representative images of anti-Cd8 staining performed on various livers cryosections from 14 m/o and 14 m/o + APAP mice. (**B**) Quantification of $Cd8^+$ cells per HPF (High Power Field). Data are represented as the mean ± S.E.M. (n = 4-6 mice per group). Statistical analysis was performed by Mann-Whitney U test.

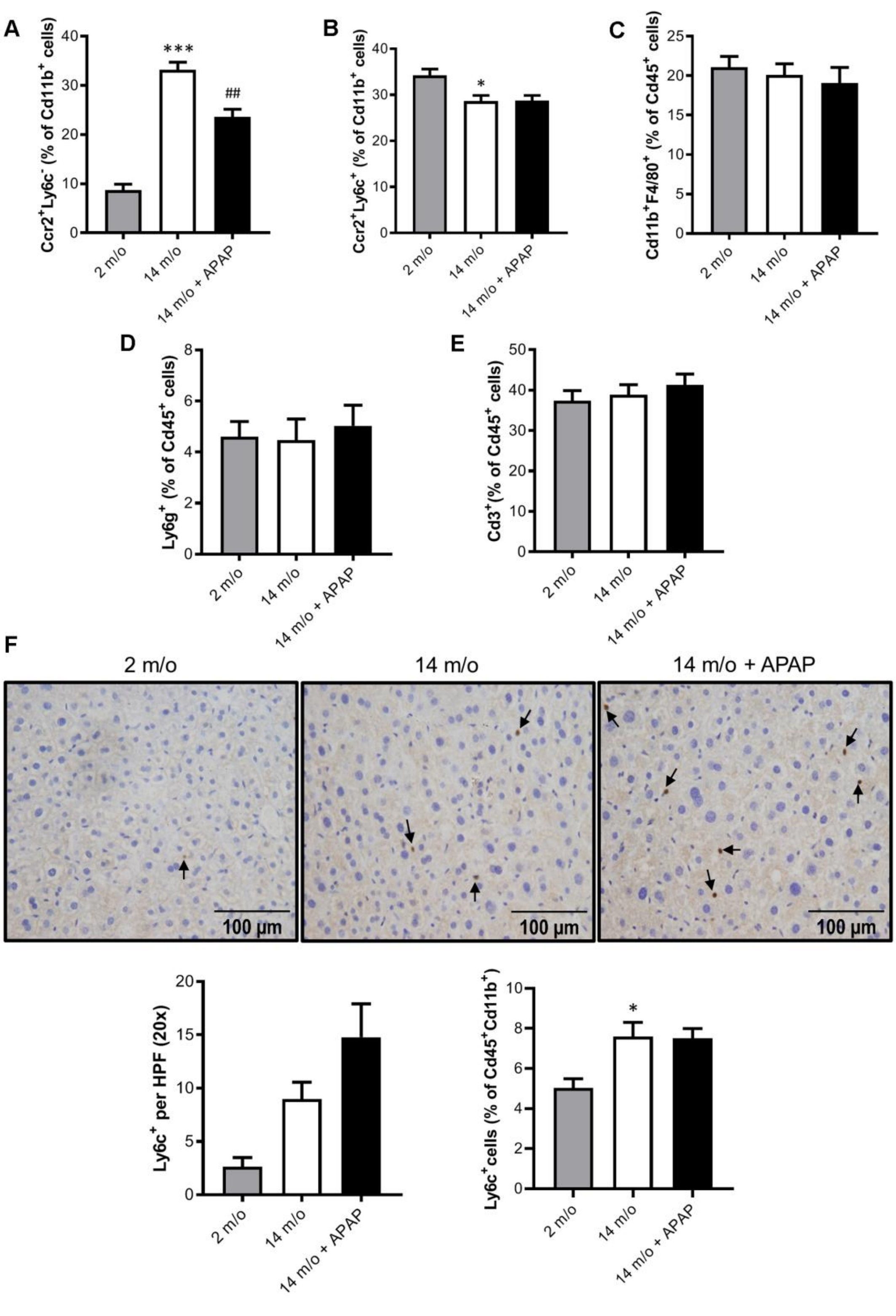

Supplementary Figure 4. Aging and chronic APAP treatment did not affect non-lymphoid populations in NPCs. Analysis of different inflammatory populations in liver NPCs from 2 m/o, 14 m/o and 14 m/o + APAP mice by flow cytometry. (**A**) Percentage of $Ccr2^{+}Ly6c^{-}$ cells from $Cd11b^{+}$ cells. (**B**) Percentage of $Ccr2^{+}Ly6c^{+}$ cells from $Cd11b^{+}$ cells. (**C**) Percentage of $Cd11b^{+}F4/80^{+}$ cells from $Cd45^{+}$ cells. (**D**) Percentage of $Ly6g^{+}$ from $Cd45^{+}$ cells. (**E**) Percentage of $Cd3^{+}$ cells from $Cd45^{+}$ cells. (**F**) $Ly6c^{+}$ per HPF (High Power Field) immunostaining (left) and analysis of total $Ly6c^{+}$ cells from $Cd45^{+}Cd11b^{+}$ cells by flow cytometry (right). Data are represented as the mean ± S.E.M. (n = 5-16 mice per group). Statistical analysis was performed by Kruskal-Wallis, one-way ANOVA or Brown-Forsythe and Welch ANOVA test followed by their respective post-hoc test. * $P < 0.05$ and *** $P < 0.001$ *vs*. 2 m/o; ## $P < 0.01$ *vs*. 14 m/o.

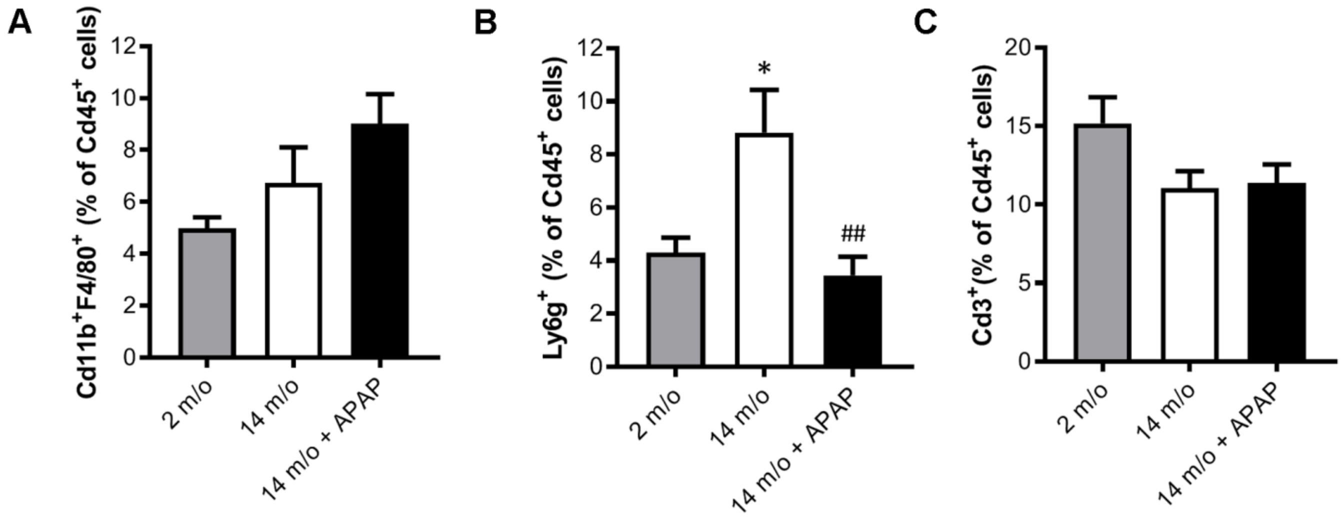

Supplementary Figure 5. Analysis of non-lymphoid inflammatory population in PBMCs from 2 m/o, 14 m/o and 14 m/o + APAP mice by flow cytometry. (**A**) Percentage of $Cd11b^+F4/80^+$ cells from $Cd45^+$ cells. (**B**) Percentage of $Ly6g^+$ from $Cd45^+$ cells. (**C**) Percentage of $Cd3^+$ cells from $Cd45^+$ cells. Data are represented as the mean ± S.E.M. (n = 9-14 mice per group). Statistical analysis was performed by Kruskal-Wallis or Brown-Forsythe and Welch ANOVA test followed by their respective post-hoc test. * $P < 0.05$ *vs*. 2 m/o; $^{\#\#}$ $P < 0.01$ *vs*. 14 m/o.

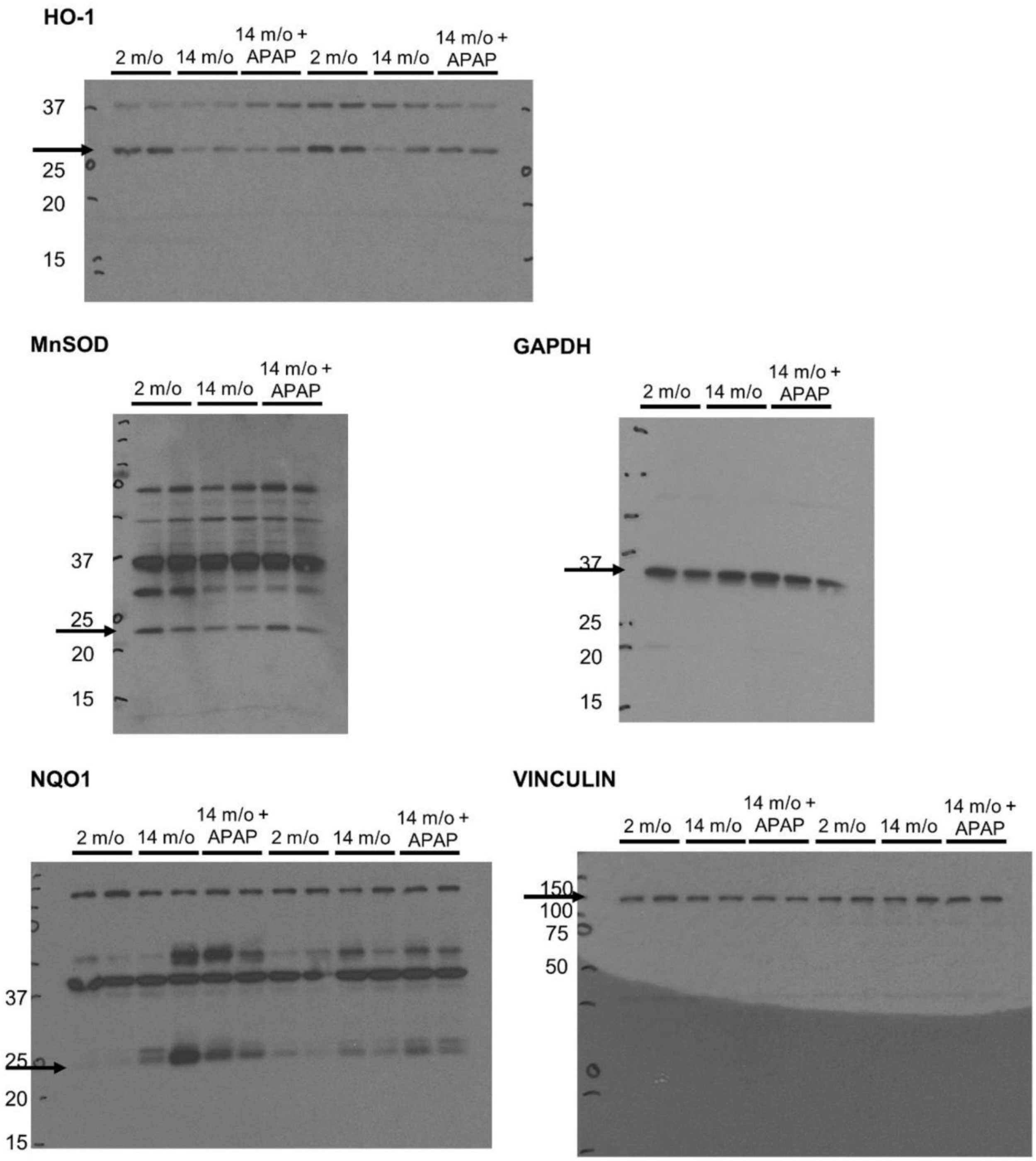

Supplementary Figure 6. Data from Figure 3A.

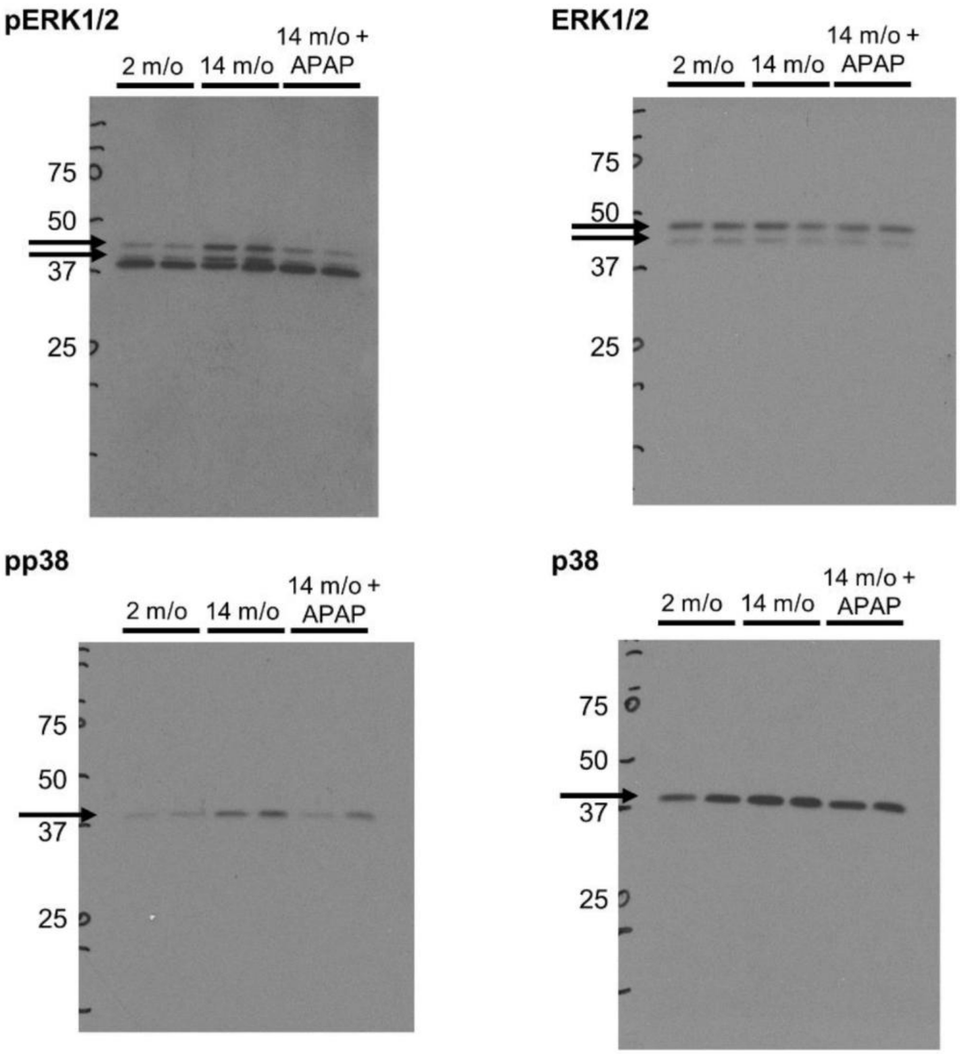

Supplementary Figure 7. Data from Figure 3E.

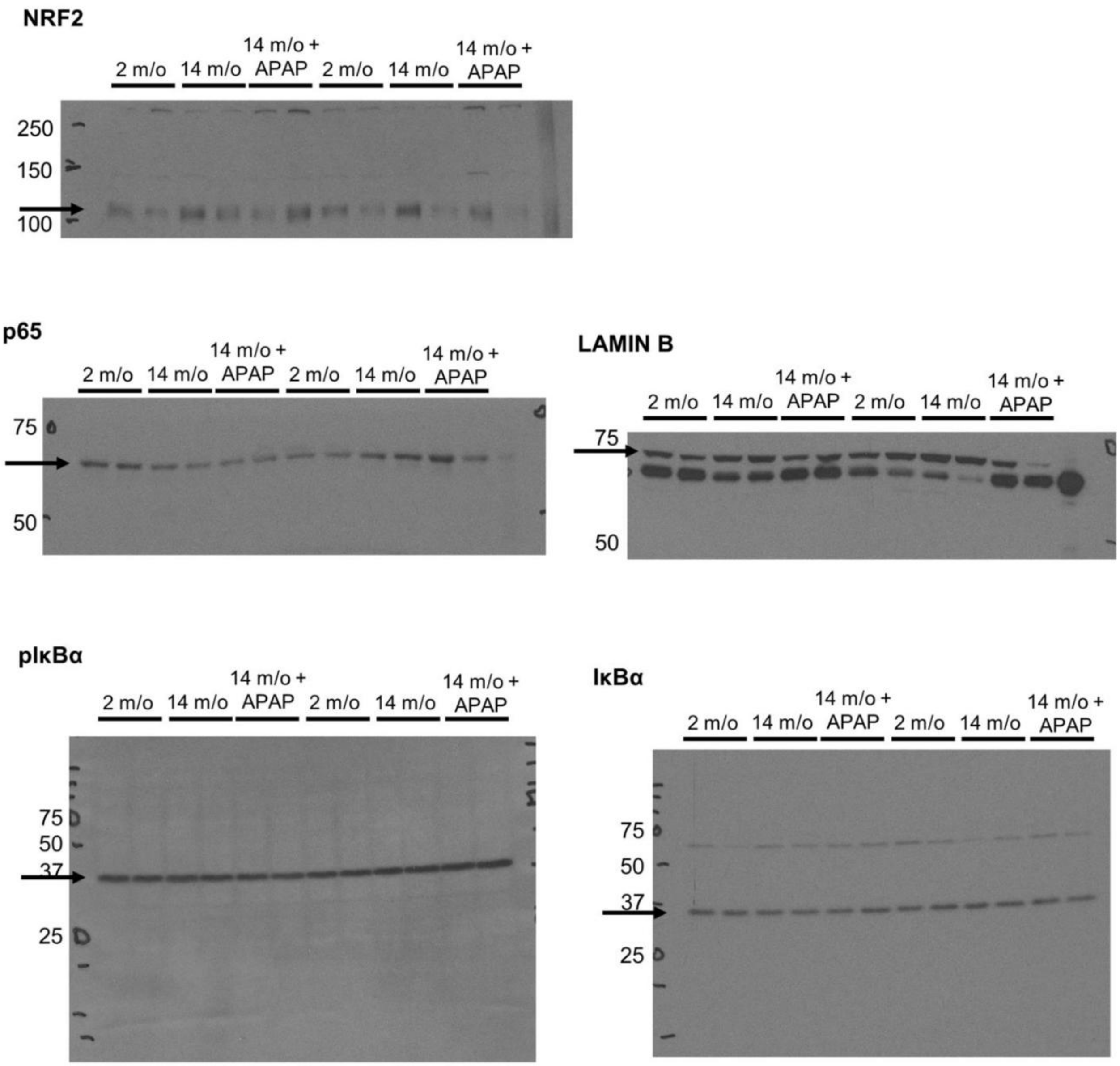

Supplementary Figure 8. Data from Supplementary Figure 2.
